# Is the non-identity problem relevant to public health and policy? An online survey

**DOI:** 10.1186/s12910-019-0379-5

**Published:** 2019-07-05

**Authors:** Keyur Doolabh, Lucius Caviola, Julian Savulescu, Michael J. Selgelid, Dominic Wilkinson

**Affiliations:** 1 Faculty of Medicine, Nursing, and Health Sciences, Monash University, Melbourne, Australia; 2 Oxford Uehiro Centre for Practical Ethics, Faculty of Philosophy, University of Oxford, Oxford, UK; 3 Department of Experimental Psychology, University of Oxford, Oxford, UK; 4 Monash Bioethics Centre, Monash University, Melbourne, Australia; 5 John Radcliffe Hospital, Oxford, UK

**Keywords:** Zika, Non-identity problem, Ethics, Survey, Harm, Person-affecting, Impersonal

## Abstract

**Background:**

The non-identity problem arises when our actions in the present could change which people will exist in the future, for better or worse. Is it morally better to improve the lives of specific future people, as compared to changing *which* people exist for the better? Affecting the timing of fetuses being conceived is one case where present actions change the identity of future people. This is relevant to questions of public health policy, as exemplified in some responses to the Zika epidemic. There is philosophical disagreement about the relevance of non-identity: some hold that non-identity is not relevant, while others think that the only morally relevant actions are those that affect specific people. Given this disagreement, we investigated the intuitions about the moral relevance of non-identity within an educated sample of the public, because there was previously little empirical data on the public’s views on the non-identity problem.

**Methods:**

We performed an online survey with a sample of the educated general public. The survey assessed participants’ preferences between person-affecting and impersonal interventions for Zika, and their views on other non-identity thought experiments, once the non-identity problem had been explained. It aimed to directly measure the importance of non-identity in participants’ moral decision-making.

**Results:**

We collected 763 valid responses from the survey. Half of the participants (50%) had a graduate degree, 47% had studied philosophy at a university level, and 20% had read about the non-identity problem before. Most participants favoured person-affecting interventions for Zika over impersonal ones, but the majority claimed that non-identity did not influence their decision (66% of those preferring person-affecting interventions, 95% of those preferring impersonal ones). In one non-identity thought experiment participants were divided, but in another they primarily answered that impersonally reducing the quality of life of future people would be wrong, harmful and blameworthy, even though no specific individuals would be worse off.

**Conclusions:**

Non-identity appeared to play a minor role in participants’ moral decision-making. Moreover, participants seem to either misunderstand the non-identity problem, or hold non-counterfactual views of harm that do not define harm as making someone worse off than they would have been otherwise.

**Electronic supplementary material:**

The online version of this article (10.1186/s12910-019-0379-5) contains supplementary material, which is available to authorized users.

## Background

### The non-identity problem

The non-identity problem has been vexing philosophers for decades. It concerns a moral question about potential people who do not yet exist, but could exist in the future. It was first set out by Derek Parfit in his 1984 book *Reasons and Persons* [1], and the problem arises when comparing actions which could improve or worsen the lives of future people. Some actions are ‘person affecting’ in that they will affect *specific* individuals in the future, while others are ‘impersonal’ since they change *which* individuals will exist in the future (for better or for worse) though they do not make specific future individuals better or worse off.

Parfit famously explored this distinction through several thought experiments. One of them, known as ‘Depletion’, asks us to choose between two policies: a ‘conservation’ policy, which rations our resources to improve quality of life gradually but steadily; and a ‘depletion’ policy, which uses all our finite resources now to boost quality of life now but with a sharp drop of quality of life in the future. Parfit points out that either policy would have a big enough impact on society to change whom people meet, and eventually have children with. As such, in 200 years’ time each policy would lead to an entirely different set of specific individuals being alive. This scenario is relevant to contemporary debates around climate change, natural resources and pollution.

**Fig. 1:**
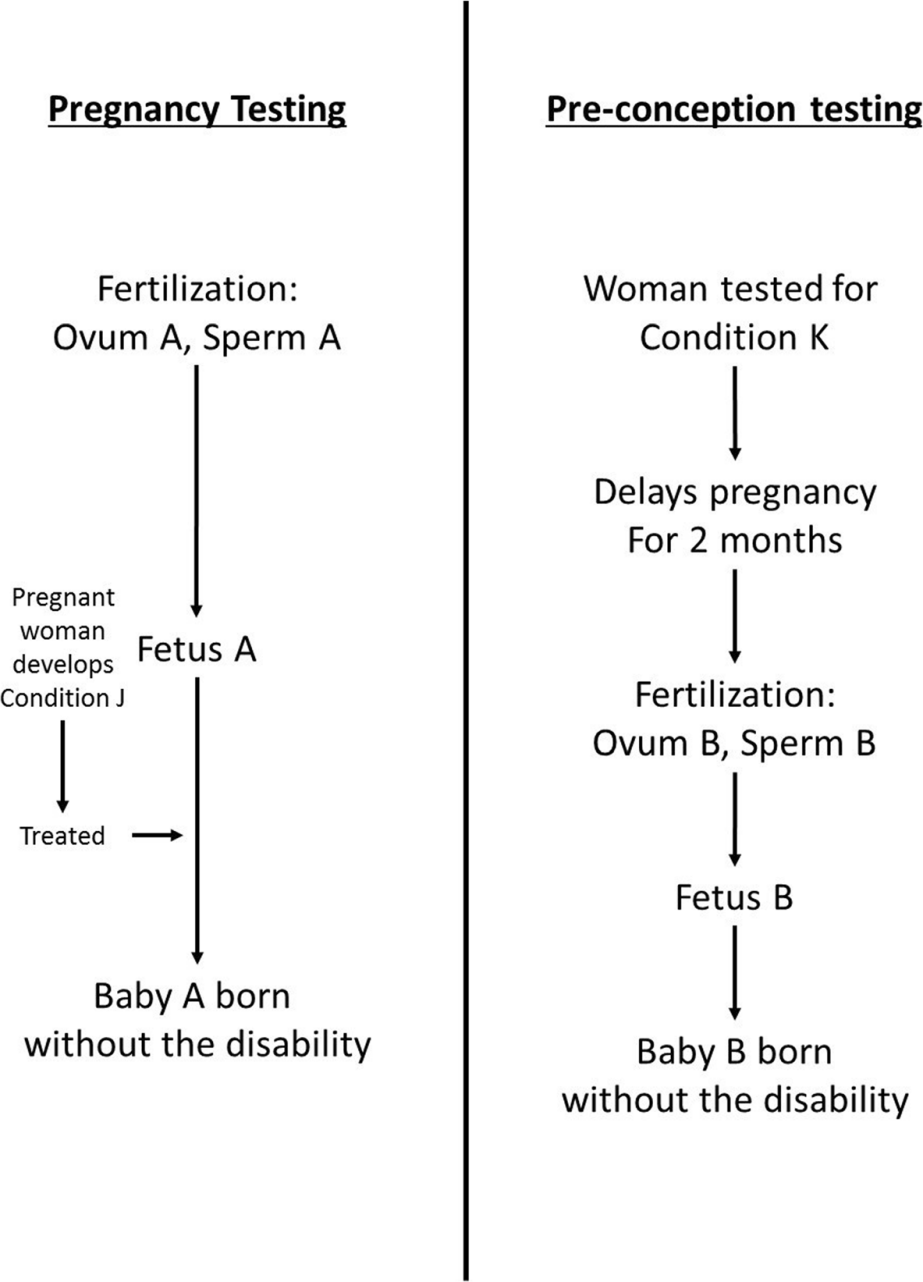
Illustration of Parfit’s thought experiment, ‘The Medical Programmes’

In another thought experiment, ‘The Medical Programmes’, Parfit imagines two diseases, Condition J and Condition K. These diseases are very mild in the people who contract them, but if pregnant women are affected by either condition, their future children develop a disability that will reduce their quality of life once they are born. We are asked to choose a medical programme to target one of the diseases. ‘Pregnancy testing’ addresses Condition J, testing for the disease and treating women if they have it – meaning their babies are born free of disability. ‘Pre-conception testing’ tests for Condition K in women who are planning pregnancies. Since Condition K cannot be cured, women with Condition K are advised to delay their pregnancy for 2 months until the disease passes. This means women screened for Condition K end up giving birth to different individuals than they would have if they had not delayed their pregnancy. Treating Condition J does not affect which specific people will be born, but simply prevents their becoming disabled, whereas treating Condition K avoids people being born with disability by ensuring different individuals will be born who are not at risk of disability. This scenario is relevant to both gene editing, and genetic selection using prenatal testing or preimplantation genetic diagnosis (Fig. 1).

In both of these thought experiments, one option is person-affecting and the other is impersonal. Intuitively, in the Depletion thought experiment it seems clear that we should favour ‘conservation’ over ‘depletion.’ But would it be wrong to choose depletion? If we choose the ‘depletion’ policy, future people could not say we harmed them or blame us for our choice, since they would not have existed if we had chosen ‘conservation.’ The impersonal choice seems hard to ethically justify, even if intuitively it seems morally better.

Similarly, there is an intuitive response that the two medical programmes are equivalent – after all, they avoid a similar number of cases of disability. However, if we do not fund the person-affecting ‘pregnancy testing,’ many people will be born disabled who could later blame us for harming them. On the other hand, if we do not fund the impersonal ‘pre-conception testing,’ then no disabled people could blame us for our choice or coherently claim we harmed them, since they would not have existed otherwise. The non-identity problem refers to the difficulty in reconciling our intuition that impersonal actions can be morally good or bad, with the fact that they do not improve or worsen the lives of any specific people.^1^

### Different views on the importance of non-identity

Views on the moral relevance of the non-identity problem can be placed into three broad categories. The first is what Parfit called the ‘person-affecting principle,’ which holds that impersonal actions have no moral weight, good or bad, because they do not help or harm any specific individuals. Accordingly, our moral decisions should only be based on person-affecting considerations. Jan Narveson supported this view. He put forward a thought experiment comparing a world with a small, hardy population virtuously striving against the elements, against a world with thriving, bustling cities and cultures. He argued that as long as the people in both worlds were similarly happy, then the worlds would be equally desirable, despite having significantly many more people in the second world [2]. Rebecca Bennett has also supported the person-affecting principle, arguing that our intuitions in non-identity cases like Parfit’s are matters of taste or preference rather than morality [3].

The second view is the ‘no-difference’ view, which Parfit defended in *Reasons and Persons*. This view holds that what matters morally is the overall wellbeing of future people, not whether specific individuals are made better or worse off. This view seems to be supported by people who feel that if society’s current energy policies would lead to future people living significantly worse lives, that would be morally wrong and socially unjust. On this view there may also be a strong moral obligation to have children who will have the best future prospects of wellbeing, since the quality of life of future children would be the key moral factor rather than the specific identity of future children. This idea that we should choose the best possible future children has been called the principle of procreative beneficence [4].

A third view is a middle position, which could be called the ‘person-affecting priority view’ [5]. Jeff McMahan has supported this view, arguing that although the impersonal effects of our actions matter morally, person-affecting considerations are weighted more heavily – impersonal benefits are good, but person-affecting benefits are better [6]. An argument for this view is that although the overall wellbeing of future people is morally important (regardless of who those people are), it would be morally preferable to improve the lives of specific people. On this view, we should choose person-affecting benefits over impersonal benefits if all else were equal, but person-affecting benefits could be outweighed if the alternative led to sufficiently greater impersonal benefits. How much greater these impersonal benefits would have to be is debatable. Table 1 below outlines how these three views might respond to Parfit’s thought experiments.

**Table 1:** The three views on non-identity in relation to Parfit’s thought experiments

View	Depletion	Two Medical Programmes
Person-affecting principle	Choose depletion, since it will not harm anyone in future and it will drastically improve people’s lives now.	Choose pregnancy testing, since pre-conception testing would not benefit any specific individuals.
Person-affecting priority	Choose depletion, unless the long-term impact on quality of life impacts under depletion are great enough or the short-term benefits are too small.	Choose pregnancy testing, unless pre-conception testing would be sufficiently more cost-effective.
No-difference	Choose conservation, since it will lead to the greatest improvement in quality of life overall. It does not matter that it does not benefit specific individuals.	The programmes are morally equivalent. If one would be more cost-effective than the other, choose that one.

### Non-identity and Zika virus disease

The recent outbreak of Zika virus disease presents a real-life instantiation of the non-identity problem that has relevance for the public health community and policy-makers [7].

Zika virus disease itself is quite mild, and infection is asymptomatic in 80% of cases. The major concern with Zika virus disease is that if pregnant women become infected with the virus, there is a risk their fetus will develop congenital Zika syndrome (CZS). CZS is a group of symptoms that often includes severe microcephaly (small head size), brain abnormalities and eventual intellectual disability.^2^ By the 4th of January 2018, there had been 223,477 confirmed cases of autochthonous cases of Zika virus disease and 3720 confirmed cases of CZS in the Americas alone [8].

There are no treatments or vaccines available for Zika and CZS yet, so many public health initiatives target the mosquito vectors responsible for most cases of Zika. This would limit the number of pregnant women whose fetuses are at risk of developing CZS. Another option for reducing the incidence of CZS is contraception. Governments could provide at-risk women with contraceptives, and advise them on how to use them to avoid or delay pregnancy. Governments might recommend delaying until the seasonal peak of the Zika transmission is passed [9], until other interventions have reduced the risk of Zika infections, or until the virus has been cleared from the area entirely. It is also worth noting the benefits of increasing access to contraceptives besides CZS, mainly through improved family planning. These benefits are particularly relevant in some of the countries most affected by the Zika virus; Latin America and the Caribbean have the highest rate of unplanned pregnancies in the world (55%), with 1 in 4 women lacking access to contraception [10].

**Fig. 2:**
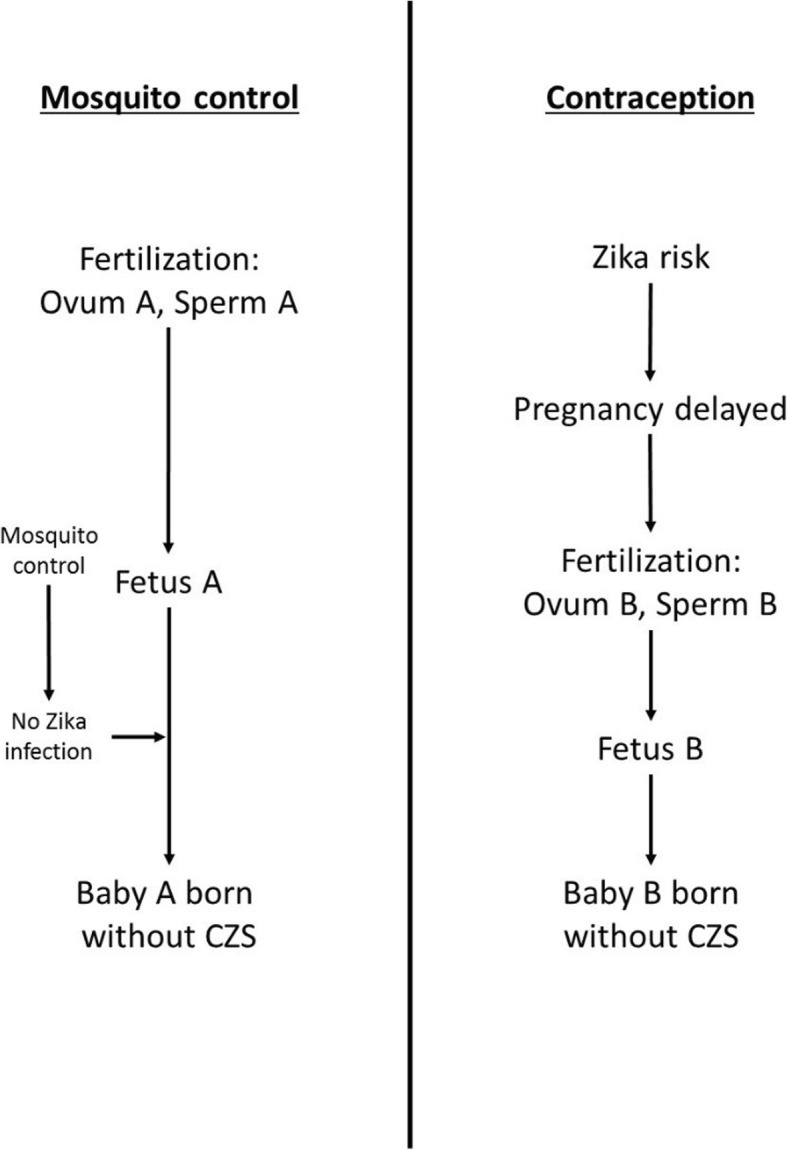
The effect on identity of mosquito control and contraception as methods to reduce the incidence of CZS

However, using contraception to reduce the incidence of CZS raises the non-identity problem: the way that it prevents CZS in a fetus is by preventing that fetus from ever being conceived, and replacing it with a genetically different one later on that is at a lower risk of developing CZS. The choice between contraception and mosquito control is remarkably similar to Parfit’s ‘The Medical Programmes’ thought experiment. Mosquito control is the person-affecting option, whereas contraception is the impersonal option. Figure 2 illustrates how these two interventions affect the identity of future people.

One difference between Zika and Parfit’s Medical Programmes is that the person-affecting benefits are identifiable in the Medical Programs, but not in the case of Zika. In the Two Medical Programmes, the programme of Pregnancy Testing would only treat those women who had Condition J, whose future children would go on to develop the disability. It is identifiable because we could know exactly which children benefitted from Pregnancy Testing. On the other hand, mosquito control would cover an entire population of women, but only prevent CZS in a subset of their pregnancies. Other pregnancies would have been unaffected even without intervention. We could not know which pregnancies, and which future people, had been helped by mosquito control – it is person-affecting, but not identifiable.

This difference also holds for the impersonal interventions: Pre-conception testing in the Medical Programmes has identifiable benefits, whereas providing access to contraception does not.

The ongoing Zika outbreak is a significant public health concern, and any ethical concerns with potential interventions against it need fairly urgent resolutions. However, philosophical analysis has not clearly resolved whether non-identity is morally relevant in the decades since it was first described. In cases like this, where there is reasonable disagreement among experts on an issue, policy should arguably take the views of the public into account. As such, it may help public health policy-makers come to a provisional stance on the non-identity problem if they understand the moral intuitions of the general public. This would also enable us to compare philosophers’ ethical analyses and the general public’s moral intuitions, and scrutinise them if they conflict. Rawls supported this approach with his argument for a ‘reflective equilibrium.’ He suggested that our normative conclusions should be based on the interaction between analysis and intuition [11]. Moreover, understanding the public’s views could help gauge whether contraception would be widely used if it were made more available, or the extent to which people might reject it on the basis of the non-identity problem or other ethical concerns.

However, at the time of writing, the only empirical data in the literature about the general public’s moral intuitions around the non-identity problem comes from a previous paper authored by ourselves. That paper performed a small-scale survey of the public’s moral intuitions around choices between impersonal and person-affecting actions. It contained questions about Parfit’s non-identity thought experiments, including some on ‘Depletion’ and some on ‘the 14-year-old girl’ (which similarly explores ideas of wrongness, harm and blame in identity-affecting decisions. See Fig. 5 in the Methods section for the full thought experiment). The survey also included questions on the choice between different interventions for addressing Zika, including mosquito control and contraception [12]. However, that earlier study did not directly determine the reasons why participants made the choices they did, and so it could not accurately determine whether non-identity influenced participants’ answers. For example, that survey could not determine if a participant preferred mosquito control over contraception because mosquito control was person-affecting, or because of other perceived benefits of mosquito control or downsides of contraception. As such, it was unable to accurately gauge how influenced participants were by the non-identity problem in their answers.

To address this knowledge gap, in this follow-up study we performed a larger online survey of a self-selected, educated sample of the general public to directly determine their views on the non-identity problem, in the context of Zika and various thought experiments. We hypothesised that:

1. Most participants would not intuitively understand the non-identity problem, or be influenced by it prior to an explanation of the problem.
2. Participants’ responses would fall into patterns that align with the person-affecting principle, the priority view, or the no-difference view.
3. The non-identity problem would play a relatively small role in participants’ decision-making compared to other ethical considerations.

## Methods

### Participants

Participants were recruited through an advertisement on the webpage of the Aeon magazine (aeon.co), an online platform that offers freely accessible articles by academics and journalists on philosophy, science and the arts (Appendix 2). They were invited to participate in a 10–15 min online survey addressing ethical issues associated with reproduction, climate, and fertility treatment. Participants were not paid for their responses. The aim was to recruit as many participants as possible through the advertisement in Aeon, with a minimum of 500.

### Procedure and materials

The survey was conducted over 2 weeks in January 2017. Participants completed the survey through Qualtrics platform (Provo, Utah), and gave informed consent at the start of the survey.

**Fig. 3:**
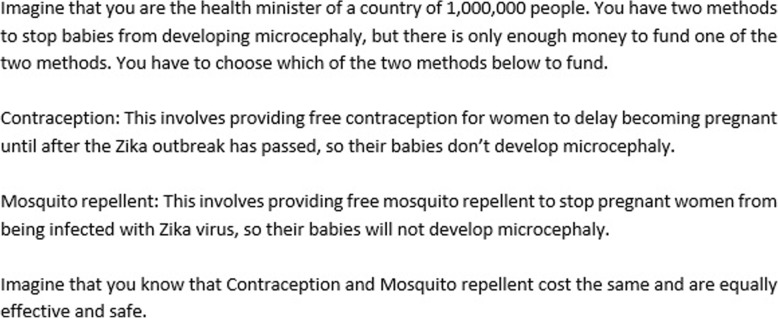
Non-identity thought experiment, focusing on the Zika virus

First, participants read an outline of the key features of recent Zika outbreaks/epidemics, and were asked to choose between reducing the burden of CZS through mosquito repellent or contraception as show in Fig. 3. Participants indicated which intervention they would prefer to fund on a 7-point scale, with a score of 1 indicating a strong preference for contraception, 7 indicating a strong preference for mosquito control, and 4 indicating no preference.

A key feature of this paper is that participants were given the option of explaining their reasons for preferring their chosen intervention as free-text answers. These questions aimed to determine whether participants intuitively understood that mosquito control would benefit specific future people whereas contraception would have impersonal benefits, and whether this made them prefer mosquito control over contraception. These questions were designed to give a more complete understanding of respondent’s views of non-identity.

**Fig. 4:**
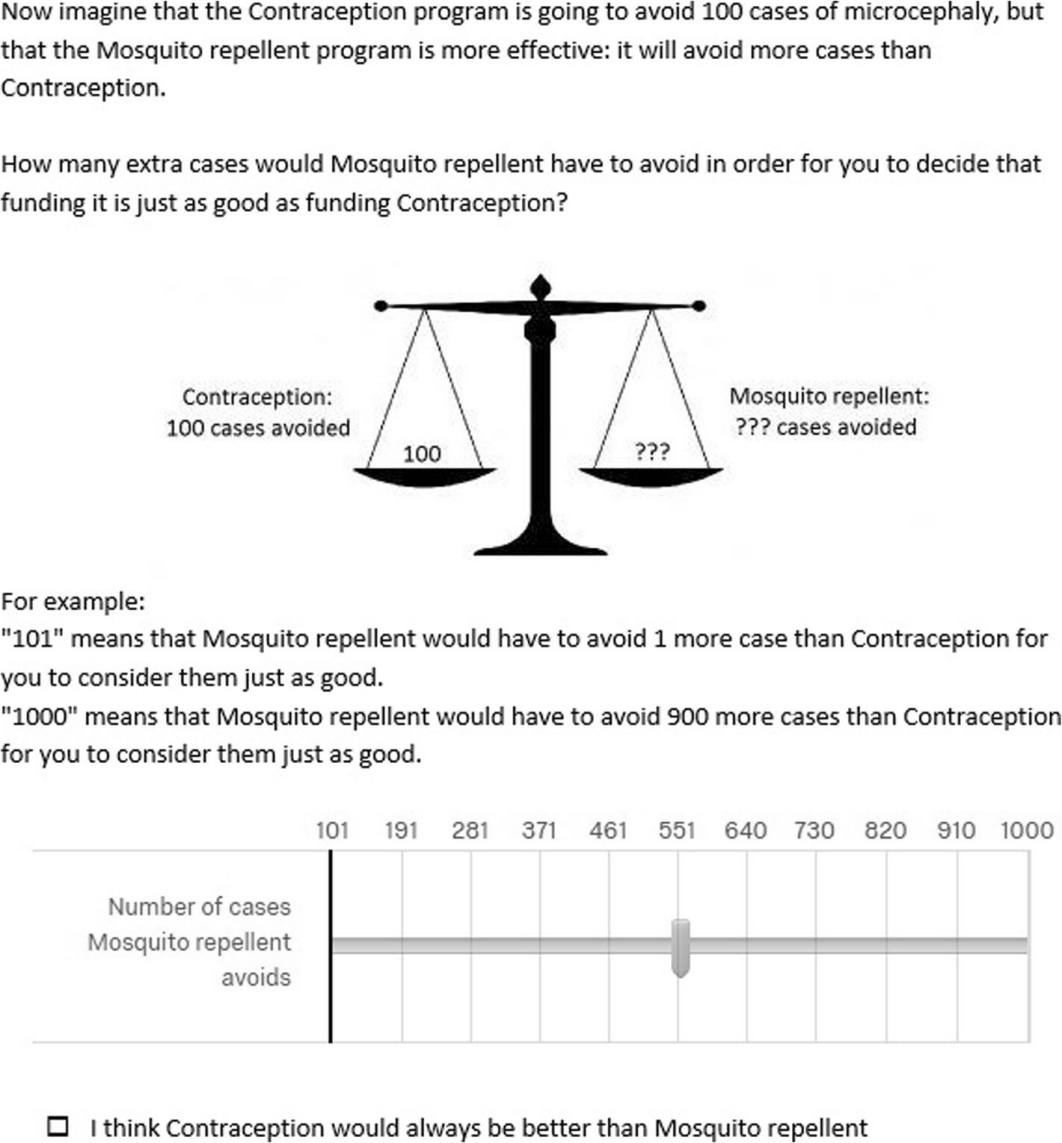
An example of the willingness-to-pay style of Zika questions, for a participant who had previously answered that they would prefer contraception over mosquito repellent

We assessed the strength of participants’ preference using a ‘willingness-to-pay’ question, which tested if participants’ preferences would change if the alternative intervention was more effective. An example of these questions is shown in Fig. 4.

After these three questions, the non-identity problem was explained to participants (Appendix 2). The three questions were repeated, to see if the explanation of the non-identity problem changed their preference for mosquito repellent or contraception.

**Fig. 5:**
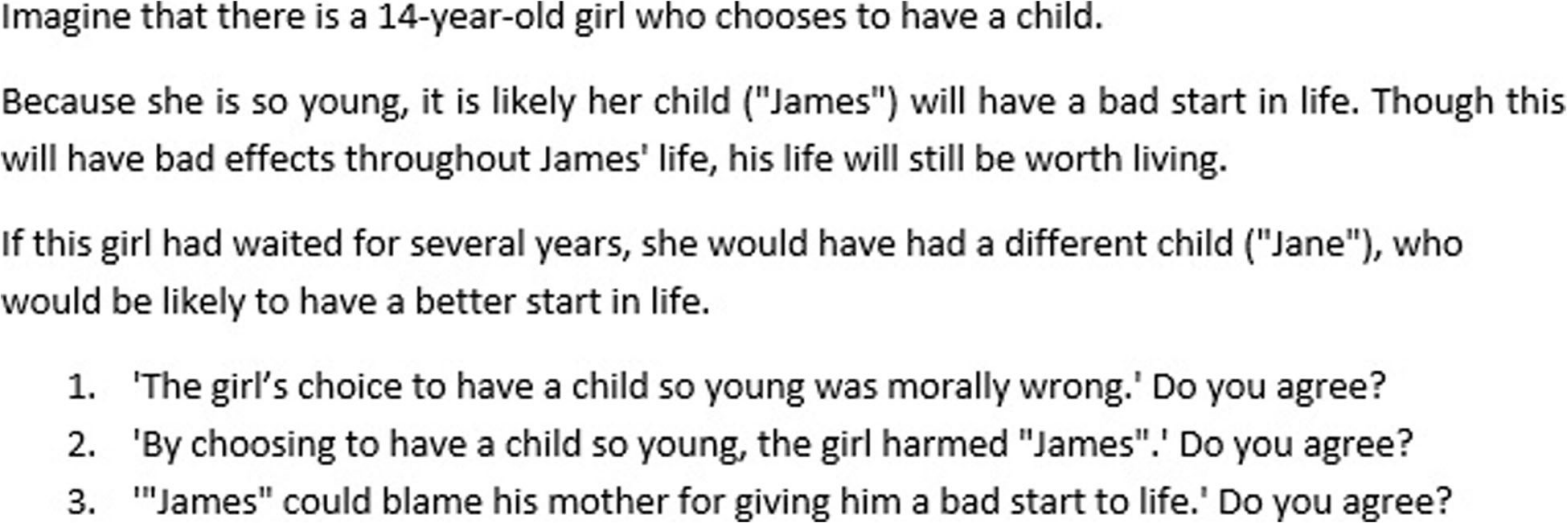
Description of Parfit’s ‘Depletion’ thought experiment, as it appeared in the survey

**Fig. 6:**
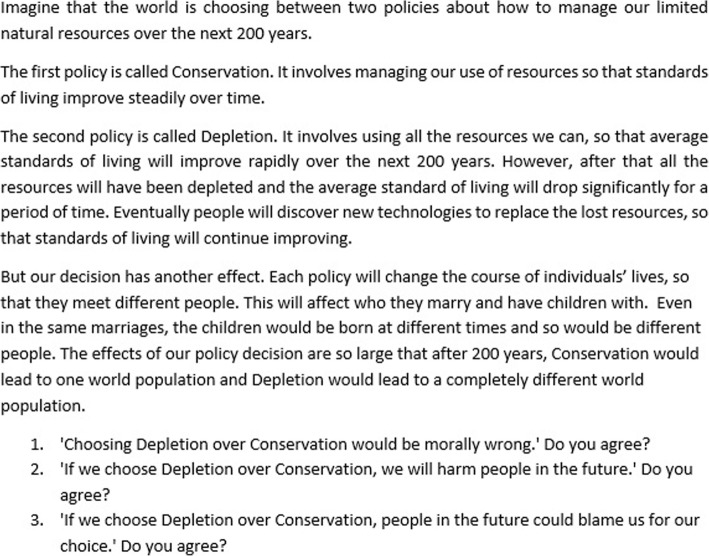
Description of Parfit’s ‘14-year-old girl’ thought experiment, as it appeared in the survey

We next presented questions about two thought experiments adapted from Parfit (the ‘14-year-old girl’ and ‘Depletion’) in order to assess participants’ *understanding* of the non-identity problem [1]. (Figs. 5 and 6 below).

For these two thought experiments, answers were recorded on a 7-point scale, from 1 (Strongly Agree) to 7 (Strongly Disagree).

**Fig. 7:**
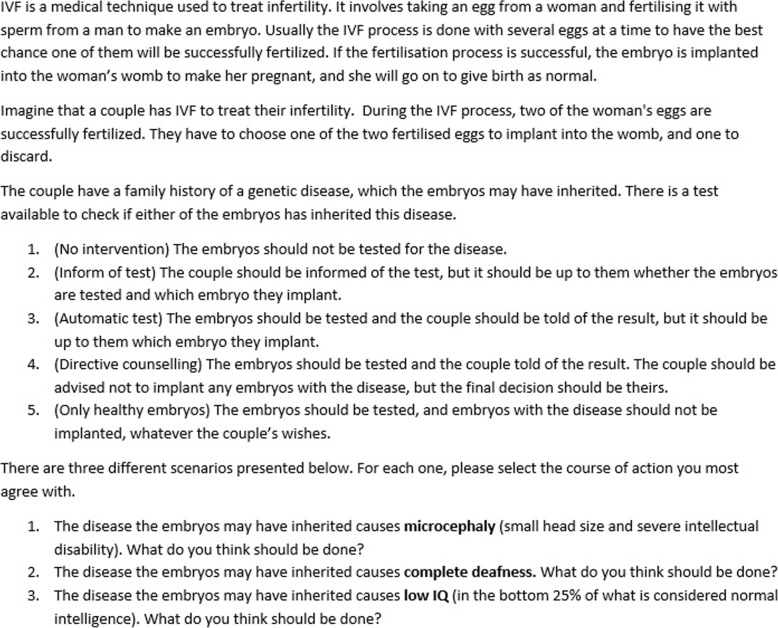
The Embryo Selection thought experiment

The final thought experiment described a scenario in which a couple uses in vitro fertilisation (IVF) to fertilise ova for a pregnancy, and two ova are fertilised, giving them a choice of which to implant. The full thought experiment is described in Fig. 7 below.

We collected basic demographic details including gender; nationality; age; highest level of education completed; past philosophy study; past reading about the non-identity problem; whether participants had children; and religiosity.

A brief summary of some of the results from the survey was previously published in Aeon magazine alongside an essay on the non-identity problem [7]. This paper provides detailed analysis of the full results, as well as ethical analysis of the findings.

### Analysis

Data was stored through Qualtrics and analysed using IBM SPSS Statistics. Individual participants’ responses were compared using paired-sample t-tests, and correlation analyses assessed associations between answers to different questions. Correlations were run between the results from the thought experiment questions and the demographics questions to determine whether certain demographic factors correlated with certain views on non-identity. A *p*-value of < 0.05 was considered statistically significant [Additional files 1 and 2].

For the free-text responses, results were grouped into categories of reasons for their preference by one of the authors. These categories were derived by two of the authors based on themes apparent in the responses, though formal textual analysis methods were not applied. They included other benefits of their chosen intervention beyond Zika, the harms of the alternative intervention, and seeing their chosen intervention as less invasive. If participants gave more than one reason, each reason was counted separately. Participants’ answers that mentioned the non-identity problem were sub-divided into those that indicated that non-identity did not influence, somewhat influenced or was the deciding factor in their answer.

## Results

### Demographics

A total of 1313 participants started the survey, but of these 550 did not finish the survey and so were excluded from the analysis, leaving a final sample of 763 (58%) responses. Table 2 sets out the demographics of the survey participants.

**Table 2:** Participants’ demographics for valid responses

Age	Median	34
	Range	18–80
Gender	Male	44%
	Female	54%
	Other	2%
Children	Yes	37%
	No	63%
Nationality	North American	44%
	European	16%
	British	10%
	Australian/New Zealander	8%
	South Asian	8%
	Other	14%
Highest level of education completed	Graduate degree	50%
	Bachelor’s degree	32%
	Attended college	12%
	High school or below	6%
Past philosophy study	Graduate	9%
	Undergraduate major	9%
	Undergraduate minor	29%
	High school	18%
	None	35%
Previous reading about the non-identity problem	Yes	20%
	No	80%
Religiosity	Religious	21%
	Atheist	46%
	Agnostic	33%

### Zika thought experiment

**Fig. 8:**
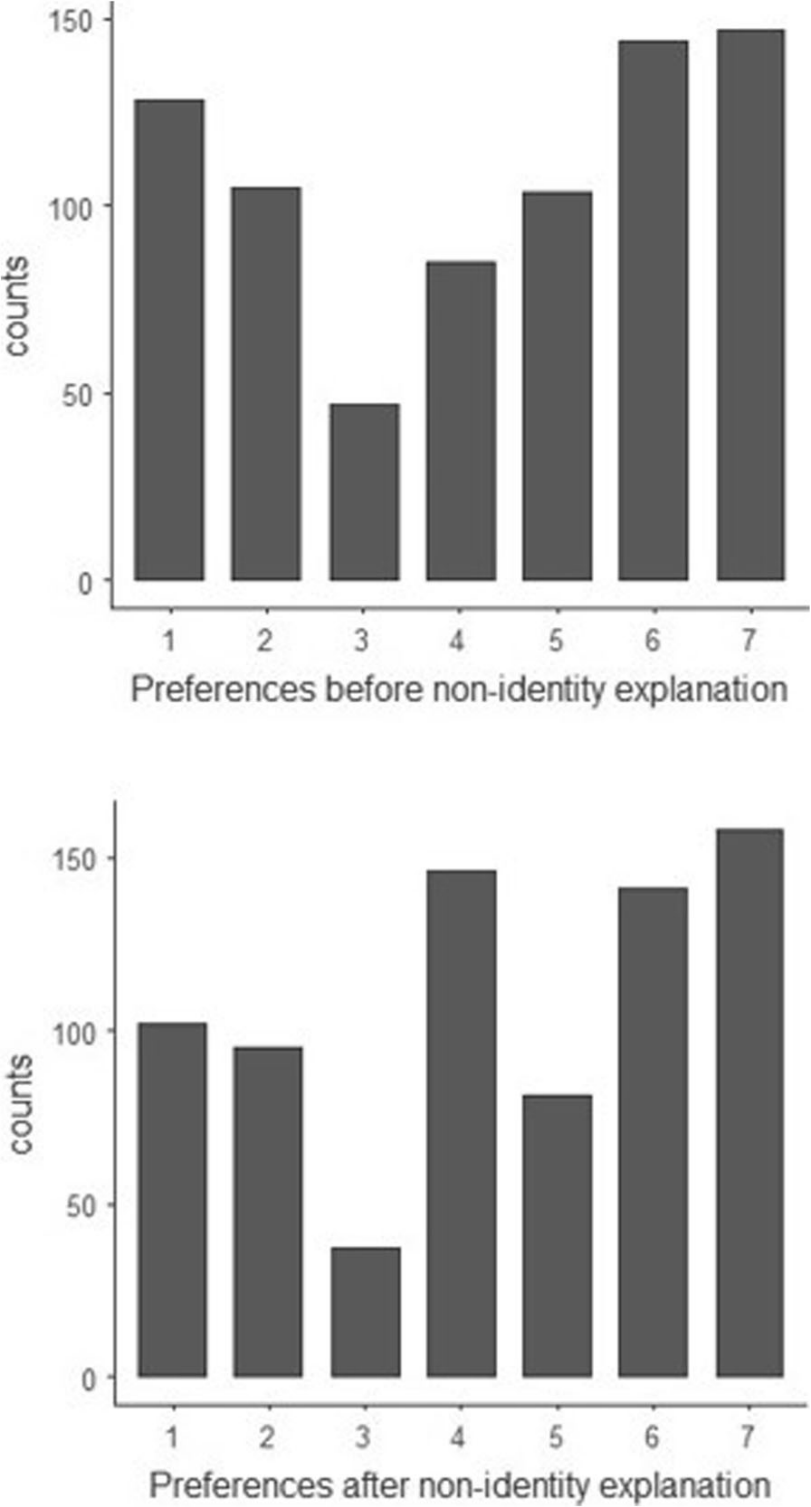
Histogram of participants’ preferences for the Zika thought experiment, before and after the non-identity problem is explained. 1 indicates a ‘Strongly prefer Contraception,’ 7 indicates ‘Strongly prefer Mosquito Repellent,’ and 4 indicates ‘No preference

On average, participants slightly preferred Mosquito Repellent over Contraception, both before the non-identity problem was explained (*M*_pre_ = 4.26, *SD* = 2.173) and after the explanation (*M*_post_ = 4.40, *SD* = 2.083). In total, 52% preferred Mosquito Repellent overall (i.e. either Strongly Preferred, Preferred or Somewhat Preferred), compared to 37% who preferred Contraception overall. There was a significant change in the mean preferences towards Mosquito Repellent after the explanation of the non-identity problem (t (762) = − 3.813*, p* < .001), which appears to be largely explained by a move from those preferring Contraception to those with no preference (11% of responses to 19%). Figure 8 below shows the full results, from 1 (Strongly prefer Contraception) to 7 (Strongly Prefer Mosquito Repellent).

**Fig. 9:**
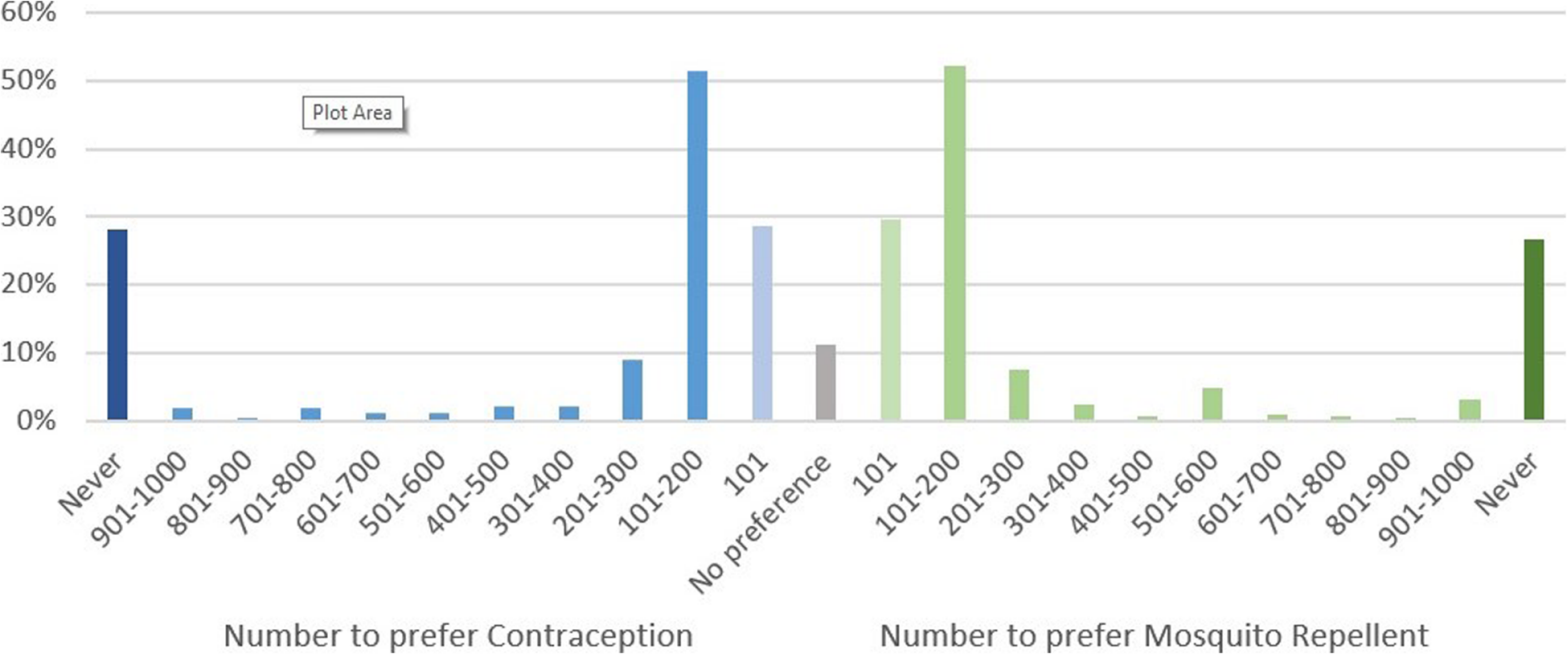
Responses to the Zika willingness-to-pay question, before the non-identity problem was explained. Indicates the proportion of respondents who would switch preference if their non-preferred intervention would avoid more cases of Zika

**Fig. 10:**
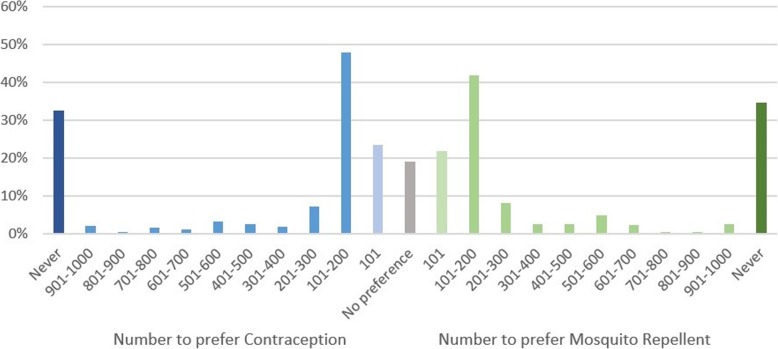
Responses to the Zika willingness-to-pay question, after the non-identity problem was explained

A minority of participants (28% before and 33% after the explanation, averaged between the two interventions), indicated that they would never change their preference even if the alternative would avoid many more cases of Zika virus. The increase in the number of participants who would never change their mind after non-identity was explained was not statistically significant. Approximately half the respondents indicated that their preference would shift if the alternative was only moderately more effective (52% before and 46% after the explanation for 100 extra cases or fewer) (Figs. 9 and 10).

In the optional free-text questions (where participants were asked to explain why they answered the way they did), 85% of all participants gave a free-text answer before the non-identity explanation and 74% did after the explanation. Once the non-identity problem had been explained, the most common reasons participants preferred contraception were believing that it would be more effective (20%), the various benefits it would have beyond addressing congenital Zika syndrome (17%), and the benefits of limiting population growth (17%). On the other hand, the most common reasons for preferring mosquito repellent were that contraception was seen as limiting reproductive freedoms (including delaying women’s pregnancies, 36%), the belief that it would be more effective (8%), and that it was seen as less invasive (8%). Appendix 3 contains more comprehensive outline of the results for this question.

Table 3 describes the most common reasons given by participants who answered they would never change their preference, while the full reasons are shown in Appendix 3.

**Table 3:** Most common reasons for participants’ responses among those who would never change their preference, both before and after the non-identity problem was explained. Reasons that were significantly associated with participants never changing their preference are marked with an asterisk, and their *p*-values are shown

	Reason given, pre-explanation	% responses (*p*-value)	Reason given, post-explanation	% responses (*p*-value)
Never prefer Contraception	Contraception limits reproductive freedoms	33%	Contraception limits reproductive freedoms	33%
	Mosquito repellent seen as more effective	13%	Mosquito repellent addresses the root cause	10%
	Personal moral qualms with contraception	9%	Mosquito repellent seen as more effective	10%
			Personal moral qualms with contraception^*^	10% (*p* ≤ .001)
Never prefer Mosquito Repellent	Contraception seen as more effective	22%	Other benefits of contraception	19%
	Benefits of limiting population growth^*^	17% (*p* = .009)	Benefits of limiting population growth	15%
	Contraception empowers women^*^	14% (*p* = .013)	Contraception seen as more effective	14%
			Health harms of mosquito repellent	14%

After the non-identity problem was explained, 32% of those who preferred Contraception and 16% of those who preferred Mosquito Repellent mentioned it in their free text response about why they had chosen their intervention. Most participants who mentioned the non-identity problem in their responses said it did not influence their decision (66% of those preferring Mosquito Repellent, 95% of those preferring Contraception).

### 14-year-old girl and Depletion thought experiments

In response to the ‘14-year-old girl’ thought experiment, on average participants were undecided on whether the girl’s choice to have a child at 14 was wrong (*M* = 4.05, *SD* = 1.81), or whether she harmed her child (*M* = 3.95, *SD* = 1.81), but they were significantly more likely to agree that her child could blame her for her choice (*M* = 3.74, *SD* = 1.78, *p* < 0.001).

**Fig. 11:**
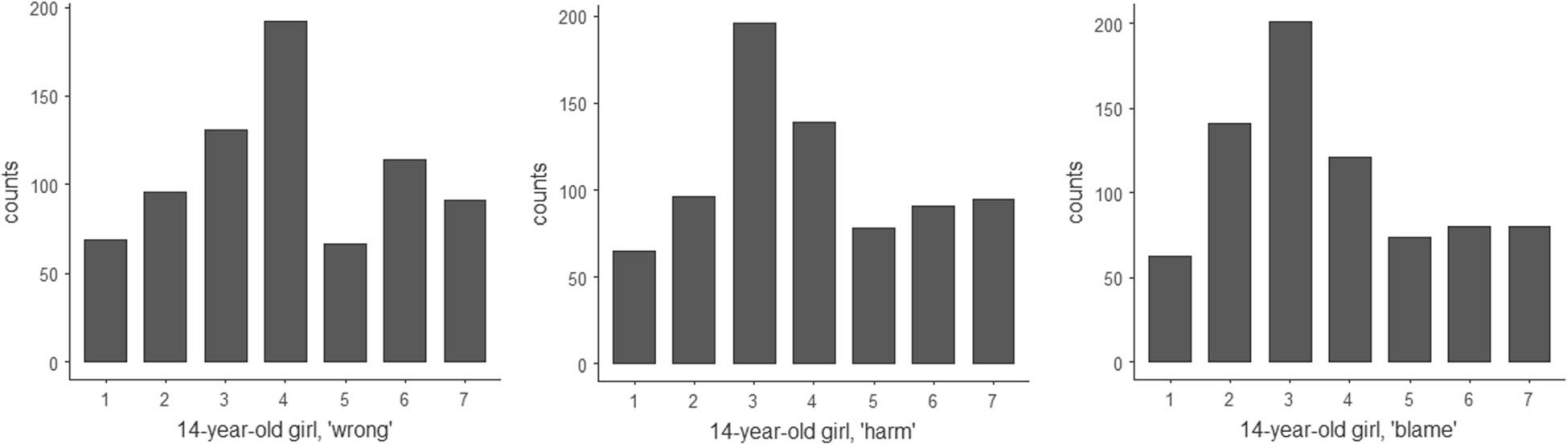
Histograms for the 14-year-old girl thought experiment, showing participants’ views on whether the girl’s choice to have a child was wrong, harmful and blameworthy. One indicates a ‘Strongly agree,’ 7 indicates ‘Strongly disagree,’ and 4 indicates ‘Neither agree nor disagree’

**Fig. 12:**
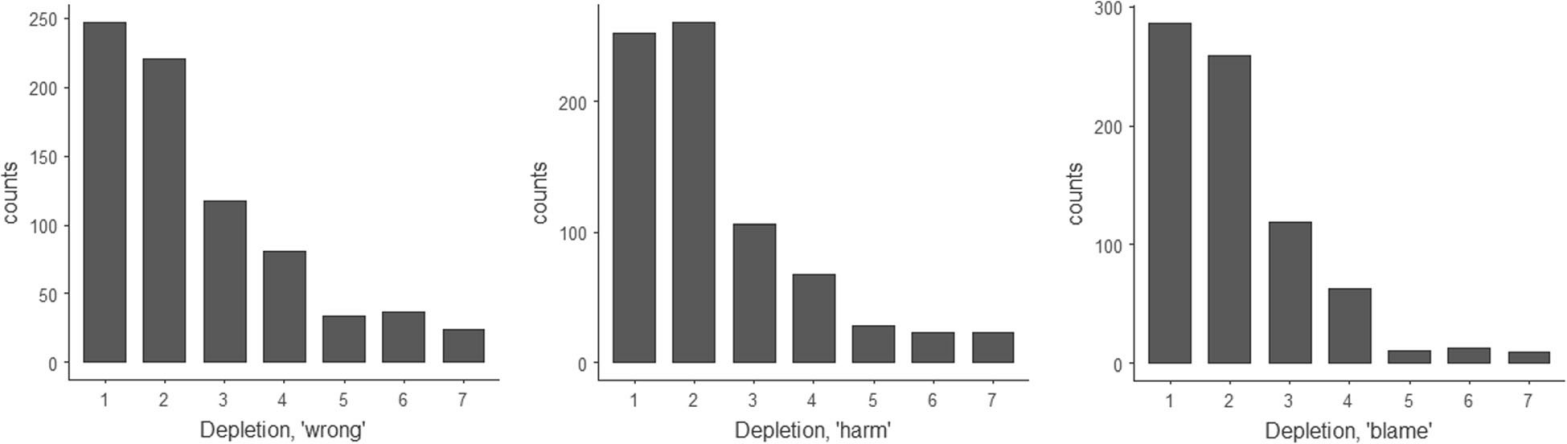
Histograms for the Depletion thought experiment, showing participants’ views on whether choosing the policy of ‘depletion’ was wrong, harmful and blameworthy. One indicates a ‘Strongly agree,’ 7 indicates ‘Strongly disagree,’ and 4 indicates ‘Neither agree nor disagree’

In the Depletion thought experiment, participants answered that choosing Depletion would be wrong (*M* = 2.53, *SD* = 1.62). They agreed more strongly that it would be harmful (*M* = 2.37, *SD* = 1.51, *p* < 0.001), and more strongly still that future generations could blame us for our choice (*M* = 2.11, *SD* = 1.25, *p* < 0.001). These views on Depletion were significantly stronger than in the 14-year-old girl questions (*p* < .001). Responses to these two thought experiments are shown in Figs. 11 and 12 below.

There was an association between participants having previously read about the non-identity problem before and disagreeing that the 14-year-old girl’s child could blame her for becoming pregnant so young (*r* = −.08, *p* = .02). Similarly, having read about the non-identity problem in the past was associated with participants denying that choosing the policy of Depletion would harm other people (*r* = −.12, *p* = .001), and that future generations could blame us for our choice (*r* = −.10, *p* = .008). This suggests that a proportion of participants could understand the non-identity problem. Conversely, studying philosophy in the past was associated with believing that future generations could blame us for choosing Depletion (*r* = .01, *p* = .01).

### Embryo selection thought experiment

In the Embryo selection thought experiment, the largest group of participants across all three disability types (microcephaly, deafness and low-normal IQ) indicated that the parents should be informed of the test (option 2, 41% average across disability types), with the second biggest group answering that the embryos should be tested, the parents should be told of the result and they should be advised not to implant any diseased embryos (option 4, 26% average). The least popular option was to not inform parents of the test at all (option 1, 6% average). (See Appendix 4 for more on the results from this question.)

Participants were more likely to be in favour of testing and embryo selection for cases of microcephaly than cases where fetuses were at risk of deafness or low IQ.

## Discussion

This study is the first large-scale systematic evaluation of non-identity intuitions through a series of philosophical thought experiments. We tested the response of an educated self-selected sample of the general public. In the Zika scenario, most participants preferred the person-affecting Mosquito Repellent, but their answers were influenced more by ethical or practical concerns other than non-identity. Participants were divided on whether the 14-year-old girl’s choice to have a child was wrong or harmful to the child, though they tended to agree the child could blame her for her choice, despite it being non-person-affecting. Most thought the policy of Depletion would be wrong, harmful and blameworthy. Finally, most participants preferred an IVF policy of informing parents of embryo testing, or of mandatory testing of embryos and advising parents not to choose diseased ones.

While there has been considerable philosophical analysis of the non-identity problem, there has been little empirical study of the degree to which identity or non-identity influence the views of the wider public. In a previous preliminary survey by the same authors [12], the majority (65%) of participants preferred person-affecting mosquito control over impersonal contraception (28%), but explaining the non-identity problem then retesting participants showed no statistically significant change in overall preferences for either intervention. The study concluded that participants most likely preferred mosquito control over contraception for reasons other than non-identity.

### Understanding of non-identity

We hypothesised that participants would fit roughly into three views of non-identity: the no-difference view, the person-affecting principle, and the person-affecting priority view. Participants could be categorised into these views based on the free-text reasons for choosing Contraception or Mosquito Control after non-identity was explained. A subset of 135 respondents explicitly mentioned non-identity in these free-text responses. Table 4 shows how participants were categorised into the 3 views, and the numbers in each category.

**Table 4:** Total number of mentions of the non-identity problem in free-text responses

View	Text response	#	%
No-difference view	Non-identity did not influence their answer	116	86%
Person-affecting priority view	Non-identity somewhat influenced their answer	5	4%
Person-affecting principle	Non-identity decisively influenced their answer	14	10%
Total mentions of non-identity		135	

The 14-year-old girl and Depletion thought experiments primarily aimed to test whether participants understood the non-identity problem the same way philosophers typically interpret it. If the 14-year-old girl chose to have a child now, or if we chose the policy of Depletion, then on standard counterfactual accounts of harm and blame the resultant future people could not say we harmed them, or blame us for their suboptimal quality of life (since they would not otherwise exist). The fact that many participants disagreed with this suggests that either they did not understand the non-identity problem, or they intuitively had a ‘non-counterfactual’ view of harm and blame [13, 14].

In his initial description of the non-identity problem Parfit characterised the concepts of harm and blame in this way: we harm a person if we make them predictably worse off than they otherwise would have been, and they could blame us for their suboptimal wellbeing only if we harmed them [1]. This ‘counterfactual’ account of harm is perhaps the most widely held view of harm [15, 16], and has been used by many philosophers who explore the non-identity problem (either implicitly or explicitly) [2, 4, 17]. Others have attempted to resolve the non-identity problem by promoting different, ‘non-counterfactual’, definitions of harm and therefore blame, that do not define harm as making someone worse off than they would have been otherwise [3, 18, 19].

While participants were fairly evenly divided on their views about the 14-year-old girl’s choice, the vast majority agreed that choosing depletion would be wrong, harmful and blameworthy. Since the two thought experiments are largely equivalent in terms of the ethics of future people, the difference in participants’ opinions are probably explained by other factors. One possible reason for the difference is that environmental conservation is a reasonably high-profile issue that many people would have already formed opinions about, whereas intentional teenage pregnancy is less visible and not necessarily an issue on which many people would already have strong views. Alternatively, the public could substantially support non-counterfactual views of harm.

### Salience of non-identity

Participants’ responses to the Zika thought experiment suggest that non-identity did not influence most of them. Although a small majority preferred mosquito control to contraception, participants’ answers in the free-text questions showed that non-identity only influenced a small group of participants. Instead, most participants answered based on other considerations, like which intervention they thought would be most effective, the benefits they would have beyond congenital Zika syndrome, and whether contraception was seen as expanding or restricting women’s reproductive freedoms. Relatively small changes in effectiveness were enough to sway the preferences of most participants, though a significant minority of respondents indicated that they would prefer either mosquito control or contraception even if the alternative would avoid many more cases of Zika (28% before non-identity was explained, 32% after).

The Embryo Selection thought experiment indirectly tested how much of a role non-identity played in participants’ moral decision-making. It did this by testing how far participants would be willing to override personal freedoms for the sake of impersonal good. It also tested how much participants would endorse the principle of procreative beneficence – the idea that out of the possible children they could have, people should choose the children who are expected to have the best lives [4]. It seems likely that participants who subscribed to the person-affecting principle would be less willing to influence the couple’s reproductive decisions, since they would see no moral reason to impinge on people’s reproductive freedom in order to choose a healthy embryo. On the other hand, those who subscribe to the no-difference view would be more likely to want to discourage or disallow implanting an affected embryo.

The results showed that most participants either thought the couple should be informed of the test (option 2), or that the embryos should be tested and the couple should be advised not to implant the diseased one (option 4). This could suggest that most participants valued the couple’s rights to be informed of the test and to make the decision, while varying on how much they thought their decision should be influenced for the sake of procreative beneficence. Participants were also more willing to discourage or disallow implanting an embryo that would develop microcephaly rather than deafness or low-normal IQ, which may suggest they were more willing to infringe on the couple’s rights in order to avoid more severe disabilities.

### Limitations

One possible limitation with the survey is that in the Zika scenario, both interventions have non-identifiable benefits (i.e. we could not know which pregnancies, and which future people, had been helped). As such, these questions may not reflect how the public would feel about Parfit’s Medical Programmes thought experiment, where both interventions did have identifiable benefits (as discussed above in the section ‘Different views on the importance of non-identity’). This is potentially quite important, since the free-text questions within the Zika scenario were particularly important in establishing what participants thought about the non-identity problem and how morally important it is. However, this difference may not have skewed responses. Since both Zika interventions had non-identifiable benefits, the main difference between them was still that Mosquito Control was person-affecting while Contraception was impersonal.

It is worth noting too, that all four scenarios are imperfect measures of the weight people place in impersonal benefit or harm, since each option intended to be impersonal could arguably lead to person-affecting outcomes. For example, in the Zika scenario, although contraception is presented as an impersonal action, it plausibly has person-affecting benefits for the women who would gain access to the contraception by helping with their family planning. Similarly, a 14-year-old girl choosing to have a child might herself be harmed by her decision to have a child at such a young age; choosing ‘depletion’ would worsen air pollution that would affect existing people’s health; and selecting an embryo with a disability could likely lead to increased healthcare costs for the family and society. But although none of the thought experiments has a truly ‘impersonal’ option, participants’ responses can still be useful. They provide insight into what participants prioritised in scenarios based on realistic, relevant non-identity cases. Moreover, the free-text responses to the Zika scenario more explicitly show how important non-identity was in participants’ decisions.

This survey was open to significant sampling bias. Since participants were recruited through an advertisement in the Aeon magazine, they were likely to have a higher educational attainment (50% had graduate degrees), were more likely to already have read content about the ethics of future people and potentially even the non-identity problem (20% of respondents). This would make them more likely than the broader public to hold views on the non-identity problem that are consistent with the views of philosophers. The fact that only a third of participants were religious also suggests that the sample may not be generalisable to the wider general public. However, that may make the results of the survey even more striking: if the non-identity problem is not thought to be a relevant consideration by a largely secular, educated sample of the general public, we might suspect that within the wider population there would be even lower weight given to non-identity.

The results of a survey like this are also very sensitive to the wording of the questions. One of the most common reasons participants gave for their preferred intervention in the Zika scenario was that they thought their preference would be more effective than the alternative. This suggests that they did not fully understand our explanation that both interventions would be equally effective and cost the same amount. More research could be done to see how the public’s preferences between person-affecting and impersonal interventions change if they genuinely appreciate that they will lead to outcomes that are otherwise equivalent. Another example is that many participants thought that choosing contraception would restrict women’s reproductive freedoms, suggesting they thought the Contraception policy would force women to delay their pregnancies rather than giving them the option to do so. Furthermore, the grouping of these free-text answers into categories was performed by a single author, raising the possibility of observer bias or errors in how answers were categorised. Formal qualitative research may help provider richer insights into how non-philosophers understand these ethical concepts and arguments.

## Conclusion

The non-identity problem poses a question that has vexed philosophers for decades. But this theoretical question has practical implications for how we approach teratogenic diseases like Zika. Our survey aimed to gather empirical data to reveal the general public’s views on the non-identity problem in an ecologically valid scenario. We chose options which were relevant to public policy, even if they imperfectly exemplified the non-identity problem – for example, it would have been more ideal to compare a treatment for Zika with contraception. The results of the survey show that participants did not attach much ethical importance to the non-identity problem. While most held either the no-difference or person-affecting priority views, the majority were influenced by other ethical or practical issues such as cost-effectiveness, practicality, and imposition on people’s freedoms.

Since the non-identity problem was first described, there has been reasonable disagreement among philosophers on its moral importance. In cases like this, it can be useful to know the general public’s views, to help policy makers decide how to address these issues. It is also striking that many people appear to hold non-counterfactual views of harm (47% in the 14-year-old girl scenario, 81% in the Depletion scenario).

Although participants had a slight preference for Mosquito Repellent in the survey, this was largely due to practical concerns rather than ethical ones, such as the belief that Mosquito Repellent would be more effective or easier to implement. That may be useful to policy makers in deciding how to approach Zika virus, or future teratogenic exposures.

It is also worth noting that the results from the other questions may be relevant to bioethics beyond the non-identity problem. The 14-year-old girl thought experiment has implications for how we address teenage pregnancy, Depletion is relevant to our approach to climate change, and Embryo Selection relates to our polices on preimplantation genetic testing in IVF. Fully exploring these issues is beyond the scope of this paper, but the results from the survey may be useful in future research.

This survey suggests that counterfactual accounts of harm and blame are at odds with the public’s moral intuitions. It may be that the general public’s intuitions around harm, blame and non-identity are misguided; however, these survey findings could also lead philosophers to critically re-examine the moral importance of non-identity or re-examine counterfactual conceptions of harm and blame.

### Additional files

Additional file 1: Raw data. (CSV 411 kb)

Additional file 2: Statistical analyses. (SPS 9 kb)

## Figures and Tables

**Fig. 13:**
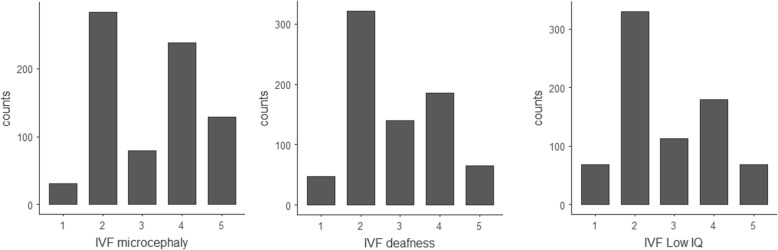
Histograms for the Embryo Selection thought experiment, showing participants’ views on the extent to which doctors should intervene when embryos are at risk of developing microcephaly, deafness or low IQ. See the Methods section for interventions corresponding to numerical value

## Data Availability

The datasets generated and/or analysed during the current study are available in the Open Science Framework repository, at: https://osf.io/8s257/ The dataset(s) supporting the conclusions of this article is (are) included within the article (and its additional file(s)).

## Appendix 1 Aeon advertisement

In a special interactive feature with the Oxford Uehiro Centre for Practical Ethics, Aeon magazine is hosting a short survey about issues around reproduction, climate, and fertility treatment. The anonymous survey asks readers to respond to a set of scenarios.

We’ll be publishing results of the survey here in a month or two, along with an essay by philosophers at the University of Oxford analysing ethical issues around identity, conception and future people.

This survey has approval from the Oxford University Research Ethics Committee, and there are no risks associated with it. Completing the survey should take approximately 10–15 min.

## Appendix 2 Explanation of the non-identity problem in the survey

Some experts have pointed out a potential difference between Contraception and Mosquito repellent. By delaying pregnancy, Contraception will change which babies exist in the future whereas Mosquito repellent does not. Imagine three scenarios:

Scenario 1: a woman is living in an area with the Zika virus. She gets pregnant with a baby girl called Tanya, but becomes infected with Zika virus. As a result, Tanya is born with microcephaly.

Scenario 2: the same woman gets pregnant with Tanya. But because she has access to Mosquito repellent, she does not get infected with Zika and her baby Tanya is born healthy.

Scenario 3: the same woman has access to Contraception instead of Mosquito repellent. She delays her pregnancy, which means when she does get pregnant it is with a different child - a baby boy called Thomas. Because she delayed her pregnancy until it was safe, she doesn’t get infected with Zika and her baby Thomas is born healthy.

So even though Mosquito repellent and Contraception both avoid cases of microcephaly, Contraception changes which babies will exist in the future, and Mosquito repellent does not.

## Appendix 3 Free-text responses to the Zika thought experiment

Tables 5 and 6 below list the reasons participants gave for preferring contraception or mosquito repellent as a means of reducing the incidence of congenital Zika syndrome. Categories are only listed if at least 1% of participants mentioned that reason.

**Table 5:** Reasons participants gave in the text-entry Zika question for preferring Contraception over Mosquito Repellent; both before and after the non-identity problem was explained

Before the explanation of the non-identity problem		After the explanation of the non-identity problem	
Reason	%	Reason	%
Believed it would be more effective	27%	Benefits beyond Zika (non-specific)	20%
Benefits beyond Zika (non-specific)	19%	Benefits of limiting population growth	17%
Benefits of limiting population growth	13%	Believed it would be more effective	17%
Health harms of Mosquito Repellent	12%	Empowering women	13%
Empowering women	12%	Environmental harms of Mosquito Repellent	11%
Improved family planning	7%	Improved family planning	10%
Environmental harms of the Mosquito Repellent	7%		
Prevention of other diseases	3%

**Table 6:** Reasons participants gave in the text-entry Zika question for preferring Mosquito Repellent over Contraception; both before and after the non-identity problem was explained

Before the explanation of the non-identity problem		After the explanation of the non-identity problem	
Reason	%	Reason	%
Contraception limits reproductive freedoms	34%	Contraception limits reproductive freedoms	36%
Believed it would be more effective	12%	Believed it would be more effective	8%
Seen as addressing the root cause of CZS	7%	Seen as less invasive	8%
Protects women already pregnant	6%	Population/economic destabilisation from Contraception	7%
Seen as less invasive	6%	Seen as addressing the root cause of CZS	7%
Protects people from Zika disease as well as fetuses	6%	Protects people from Zika disease as well as fetuses	6%
Benefits beyond Zika (non-specific)	5%	Protects women already pregnant	4%
Others’ moral qualms with Contraception	4%	Better for older/subfertile women wanting children	4%
Population/economic destabilisation from Contraception	4%	Personal moral qualms with Contraception	4%
Personal moral qualms with Contraception	3%	Benefits beyond Zika (non-specific)	4%
Uncertainty about how long pregnancies must be delayed	3%	The non-identity problem	4%
Contraception already available/used	2%	Contraception already available/used	2%
Simplicity of use	2%	Uncertainty about how long pregnancies must be delayed	2%
Health harms of Contraception	2%	Protects those women not expecting to get pregnant (young girls, rape victims etc.)	2%
Better for older/subfertile women wanting children	2%		
Protects those women not expecting to get pregnant (young girls, rape victims etc.)	2%		

## Abbreviation

CZS: Congenital Zika syndrome

## Footnotes

1 Throughout this paper, ‘non-identity’ and ‘the non-identity problem’ are used interchangeably, referring to the question of whether there is a moral difference between person-affecting and impersonal actions. It is also worth pointing out that non-identity is relevant for both improving and worsening the lives of future people, although for simplicity’s sake this paper often refers to one or the other.

2 Like several other infectious diseases, Zika virus disease also carries a small risk of Guillain-Barre syndrome developing in infected people.

## References

[1] Parfit D (1984). Reasons and persons.

[2] Narveson J (1973). Moral problems of population. Monist.

[3] Bennett R (2009). The fallacy of the principle of procreative beneficence. Bioethics..

[4] Savulescu J (2001). Procreative beneficence: why we should select the best children. Bioethics..

[5] Wilkinson D, Schaefer GO, Tremellen K, Savulescu J (2015). Double trouble: should double embryo transfer be banned?. Theor Med Bioeth.

[6] McMahan J (2013). Causing people to exist and saving People’s lives. J Ethics.

[7] Wilkinson D, Doolabh K. Which lives matter most? Aeon. Aeon Media Group Ltd; 2017. https://aeon.co/essays/should-we-take-ethical-account-of-people-who-do-not-yet-exist. Accessed 9 Jul 2017

[8] Pan American Health Organization/World Health Organisation (2018). Zika suspected and confirmed cases reported by countries and territories in the Americas cumulative cases, 2015–2017.

[9] Martinez ME (2016). Preventing Zika virus infection during pregnancy using a seasonal window of opportunity for conception. PLoS Biol.

[10] Bahamondes L, Ali M, Monteiro I, Fernandes A (2017). Contraceptive sales in the setting of the Zika virus epidemic. Hum Reprod.

[11] Rawls J (1971). A theory of justice.

[12] Doolabh K, Caviola L, Savulescu J, Selgelid M, Wilkinson DJC (2017). Zika, contraception and the non-identity problem. Dev World Bioeth.

[13] Kahane G, Savulescu J (2012). The concept of harm and the significance of normality. J Appl Philos.

[14] Williams NJ, Harris J (2014). What is the harm in harmful conception? On threshold harms in non-identity cases. Theor Med Bioeth.

[15] Feinberg J (1984). Harm to others. The moral limits of the criminal law.

[16] Purshouse C (2016). A Defence of the counterfactual account of harm. Bioethics..

[17] Arrhenius G (2005). The person-affecting restriction, comparativism, and the moral status of potential people. Ethical Perspect.

[18] Harman E, Roberts MA, Wasserman DT (2009). Harming as causing harm. Harming future persons: ethics, genetics and the nonidentity problem.

[19] Shiffrin SV (1999). Wrongful life, procreative responsibility, and the significance of harm. Legal Theory.

